# Silver Nanoclay Human Albumin Serum Composite Electrochemical Biosensor for Quantification of Zidovudine in Tablets

**DOI:** 10.1007/s11095-025-03954-9

**Published:** 2025-11-11

**Authors:** Sapokazi Timakwe, Mangaka C. Matoetoe

**Affiliations:** Chemistry Department, Cape Peninsula University of Technology, Symphony Way, P.O. Box 1906, Bellville, 7535 South Africa

**Keywords:** biosensors, human serum albumin, silver nanoclay composites, zidovudine

## Abstract

**Objective:**

This work aims to develop an electrochemical biosensor for the analysis of zidovudine (ZDV) in commercial tablets.

**Method:**

The biosensor utilised silver nanoclay composites (AgNCs) together with drop-coated Human Serum Albumin (HSA) on a glassy carbon electrode (GCE/AgNCs/HSA). The electrochemical properties of the GCE/AgNCs/HSA were studied using cyclic voltammetry (CV) and analysed using the Randles–Sevcik equation. Procedurally, optimised differential pulse voltammetry (DPV) technique in 1 M PBS, pH 7.04, was used to determine analytical parameters of the methods, validation, and study of commercial tablets.

**Results:**

The optimum procedural conditions used were -0.4 V to -1 V potential range, 0.0405 V step potential, 0.075 V modulation amplitude, 0.01 s modulation time, and 0.25 s interval time. GCE/AgNCs/HSA film’s electrochemical properties obtained were a diffusion coefficient (D) of 1.55 × 10–11 cm2.s-1, a heterogeneous rate constant (Ks) of 3.40 × 10–6 cm.s-1, with two electrons. The calibration graph linear range was from 0.12 to 6.98 μM with a low limit of detection (LOD) of 0.3 μM, and 1.0 μM limit of quantification (LOQ). Method validation showed an acceptable %RSD for reproducibility and repeatability. The sensor was stable over 10 days and remained unaffected by the presence of the interferences studied. The commercial study of 300 mg commercial tablets was found to be 301.3 mg with 1.9% RSD, which is in agreement with the tablet's stated amount.

**Conclusion:**

The development of the GCE/AgNCs/HSA biosensor was successful and efficiently used to quantify ZDV in tablets.

**Graphical Abstract:**

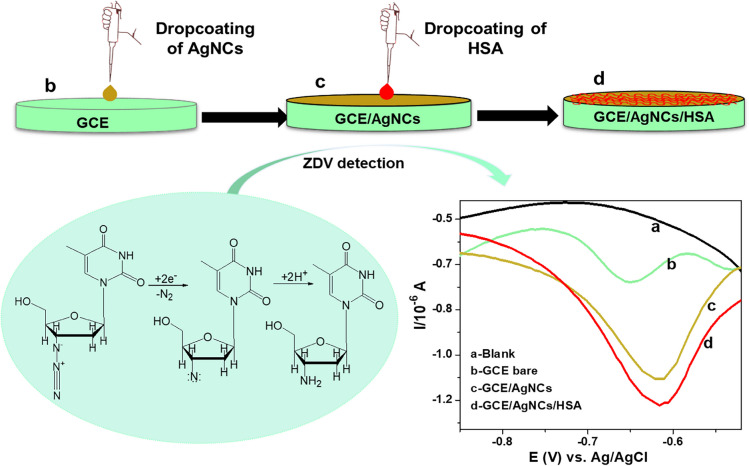

## Introduction

Effective management of human immunodeficiency virus/acquired immunodeficiency syndrome (HIV/AIDS) is achieved through diagnosis, treatment, prevention and access to good health care. Thus, good quality antiretroviral drugs (ARVs) and adherence to treatment is mandatory [1]. Zidovudine (ZDV) is a type of nucleoside reverse transcriptase inhibitor (NRTI) ARV drug also used in inhibition of mother-to-child spread [2]. Large consumption of ARVs resulted in them being environment contaminants, with ZDV having little therapeutic interval that is, becoming ineffective to many patients causing health complications [2]. Therefore, this has brought attention in the investigation and monitoring of the drug quality. The common, monitoring of the drug is achieved with techniques such as chromatographic [3], voltammetric [4, 5] and spectroscopic [5, 6]. The disadvantages of these techniques is usage of expensive instruments, laborious sample preparation, longer analysis time that the methods require [7, 8], various chemical interferences that affects the accuracy of the results, as well as possible analyte loss during sample preparation process limits the application of these techniques. Hence, development of electrochemical analysis methods using which are sensitive, stable, fast analysis time, and low-cost [8–10]. These techniques application are enhanced by using biosensors. The electrochemical biosensors simultaneously determines drugs, food additives, and environmental contaminants with one electrochemical sensor [11]. Biosensors consists of a biosensitive layer made up of biological elements attached covalently to the transducer [11–13]. A transducer generates the response resulting from the interaction of the analyte with the biomolecule and convert it into a detectable signal, followed by a chemically selective layer which separates the analyte response from its instant environment [14, 15].

The electrochemical biosensor fabrication involves combination of transduction elements such as redox pairs, metallic nanomaterials, carbon nanostructure for electron transfer efficiency, together with biological recognition elements such as deoxyribonucleic acid (DNA), enzymes, protein, Ribonucleic acid (RNA), antibodies, aptamers [14]. Nanomaterials electrode modification increased surface area, immobilisation efficiency, catalytic activity of proteins and other biological compounds thus increasing the lifespan of the biosensor as a result of improved analyte mobility of analyte, biocompatibility, chemical stability and good adsorption capacity [9, 16, 17].

Hence, the above advantages were combined with the nanoclay’s low cost and ease of functionalisation with conducting chemicals (silver) to increase conductivity [17–19]. Other nanoclay composites’ desirable properties are robustness as a result of poor chemical and mechanical reactivity. Its widespread applicability in sensors is due to their poor permeability, swelling ability, large surface area/volume ratio, biocompatibility, low toxicity as well as their intrinsic adherence to their metal dopants. They also have high ion sorption/exchange capability [20, 21]. The ion exchange process in combination with the layered structure facilitates covalent bonding of the modifier to atoms on the nanoclay sheet [21] An incorporation of human serum albumin (HSA) protein that is known to easily bound on nanoparticle surfaces, enhancing their capacity to interact with a wide range of ions and small molecules thus improving the biosensor sensitivity [8, 20, 21] was adopted. A composite of silver nanoclay coated with HSA acting as a protective layer prevents silver nanoparticles from aggregating, resulting in improved stability of the biosensor [22, 23]. Electroanalysis of ZDV has been achieved with various nanomaterials [24, 25]. Very few articles study sensor’s behaviour to pharmaceutical dosage forms. In this study, electrochemical sensor effectively fabricated using silver nanoclay composite and HSA was developed. The developed biosensor GCE/AgNCs/HSA show catalytic activity towards the electrochemical reduction of ZDV which has been studied by cyclic voltammetry (CV) and used in quantifying ZDV by differential pulse voltammetry (DPV) in biological samples and commercial ZDV tablets.

## Experimental

### Reagents

Silver nitrate, AgNO_3_ (≥ 99.0%), trisodium citrate dihydrate (TSC), Na_3_C_6_H_5_O_7_.2H_2_O (≥ 99.0%), surface modified nanoclay (NC) (35–45 wt.%), sodium hydroxide pellets, and hydrochloric acid (HCl) were bought from Sigma Aldrich (UK) and used without purifying further. MilliQ water from the Millipore system was used throughout the experiment. ZDV reference standard (≥ 98.0%) and HSA (≥ 99.0%) were purchased from Sigma Aldrich (US), including potassium dihydrogen phosphate (KH_2_PO_4_) (≥ 99.0%), disodium hydrogen phosphate (Na_2_HPO_4_) (≥ 99.0%), Dextrose Glucose (≥ 99.5%), citric acid (≥ 99.5%), and ascorbic acid (≥ 99.0%). The ZDV commercial tablets (300 mg) were obtained at District Six Clinic (Cape Town, SA).

### Solution Preparation

All solution preparations were done using MilliQ water. ***Preparation of Phosphate buffer solution (PBS)*** different concentrations (0.01, 0.1 & 1 M) were prepared and adjusted to pH 7.04 with 1 M NaOH and 1 M HCl and stored at room temperature. ***HSA Solution*** was achieved by diluting 1 mg/mL HSA in 5 mL of 0.1 M PBS (pH 7.04) resulting in a final concentration of 0.2 mg/mL which was refrigerated at 2 ℃, as per the packaging instructions. **ZDV solution** of 0.1869 mM was prepared and stored in a brown bottle and refrigerated.

### Silver Nanoclay Composites Synthesis

The silver nanoclay composites (AgNCs) were prepared by mixing 0.5 g of nanoclay (NC) and 1.0 mM silver nitrate solution. The mixture was heated to boil while stirring for 20 min at 240 ℃, 265 rpm, followed by the slow addition of 4 mL, 0.01 M of TSC. The mixture was further heated for another 15 min and cooled at room temperature, followed by purification with MilliQ water through centrifugation.

### Spectroscopic Characterisation and Electrochemistry Procedures

Fourier Transform Infrared Spectroscopy (FTIR) characterisation was done using Perkin Elmer spectrometer (USA) between the wavenumber 3700 cm^−1^ to 700 cm^−1^. Firstly, the background noise was measured and subtracted to remove unwanted responses followed by the scanning of the sample by compressing the sample onto the sample holder while recording the spectrum. A multipurpose X-ray diffractometer (XRD) D8-Advance from BRUKER AXS (Germany), operated in a continuous scan in locked coupled mode with Cu-Kα radiation was utilised for XRD measurements. The samples were mounted in the centre of the sample holder on a glass slide and levelled up to the correct height. The measurements were scanned within a 10 to 80 2-theta range, with a typical step size of 0.034° in 2-theta. A position sensitive detector, Lyn-Eye, was used to record diffraction data at a typical speed of 0.5 s/step which is equivalent to an effective time of 92 s/step for a scintillation counter. Autolab PGSTAT 101 (Metrohm, SA) was for electrochemical measurements with a software NOVA 2.1. All measurements were done at room temperature (25°C) with glassy carbon electrode (GCE) as a working electrode (A = 0.071 cm^2^), platinum wire as counter electrode and Ag/AgCl (3 M KCl) as the reference electrode. The cleaning of the working electrode was done with alumina slurry of 1.0, 0.3 and 0.05 µm on a polishing pad, followed by 5 min sonication of GCE in water and ethanol between polishing steps. Electrochemical measurements were achieved in 3 mL solution of 0.1 M HCl and PBS supporting electrolyte which should be deoxygenated with nitrogen gas before measuring. The pH metre HANNA HI 2210 (SA) was used to adjust the pH.

### Sensor Fabrication Method Optimisation

The dropcoating method of HSA involved modification of GCE with 5 µL AgNCs and left to dry for 26 min, followed by dropcoating of 2 µL HSA onto dried GCE/AgNCs electrode and further dried for 15 min before the measurements. In the case of HSA immersion method, the freshly prepared and dried GCE/AgNCs electrode was left in the HSA solution for 15 min. The prepared GCE/AgNCs/HSA were used to detect ZDV.

#### ZDV Method Detection Optimisation

The best method for detecting ZDV with the developed GCE/AgNCs/HSA sensor was determined by varying one parameter, while keeping others constant. These parameters include drying time of HSA, effect of electrolyte stirring time and technique optimisation such as step potential, modulation amplitude, modulation time and Interval time.

#### Reproducibility, Repeatability, Stability, Interferences, and Recovery Studies

The reproducibility of GCE/AgNCs/HSA sensor was assessed in 1 M PBS, pH 7.04 with 3.24 μM ZDV using DPV. Three replicates of the ZDV current response were measured for repeatability purposes. To monitor the stability of the sensor, the GCE/AgNCs/HSA sensor was prepared and used to measure the initial ZDV response. Then, the sensor was stored in PBS solution and was used to monitor the current response of ZDV after every two days for a period of ten days i.e. 2, 4, 6, 8 and 10 days.

The solutions of 18.69 mM D-Glucose, ascorbic, and citric acid were assessed as possible interferants as they are commonly used as supplements for patients with HIV. The ratios 1:1, 1:10 and 10:1 (ZDV: Interferent) were assessed in 1 M PBS, pH 7.04 at GCE/AgNCs/HSA. The interference studies were also done in the presence of other ARVs, where the solutions contained 2.21 and 2.25 mM of efivarenz and nevirapine. Recovery in spiked urine samples was carried out using three ratios of Urine:1 M PBS (i.e. 1:4, 1:9 and 1:14) which were spiked with ZDV solution to measure ZDV response at GCE/AgNCs/HSA. The electrode was cleaned constantly prior to each measurement as per Section "Spectroscopic Characterisation and Electrochemistry Procedures".

#### Analysis of Commercial Tablets

Three ZDV commercial tablets were weighed separately and dissolved in Milli-Q water. The solutions were allowed to stand for few hours for complete dissolution. The triplicate volume of 100 µL of each solution was pipetted into 1 M PBS electrolyte, pH 7.04 and the ZDV analysis was carried out with the GCE/AgNCs/HSA film, using optimum conditions: 0.0405 V step potential, 0.075 V modulation amplitude, 0.01 s modulation time, and 0.25 s interval time.

## Results and Discussion

### Characterisation of Silver Nanoclay Composites

**Fig. 1:**
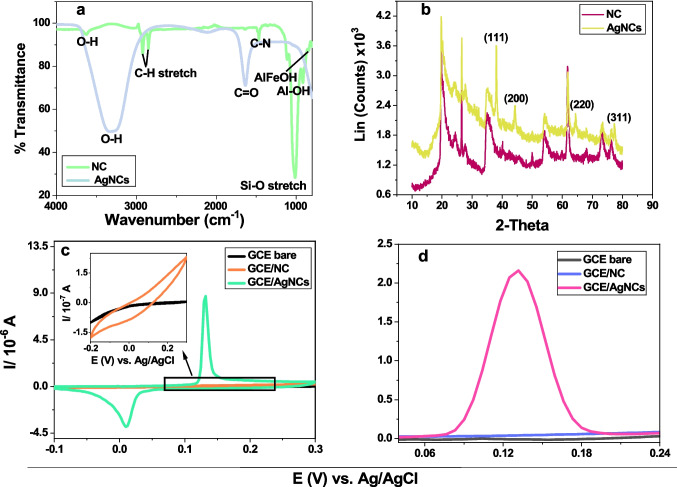
Nanoclay (NC) and silver nanoclay composites AgNC (**a**) FTIR (**b**) XRD (**c**) CV at 0.08 V/s, & silver nanoclay composites (**d**) DPV at potential step 8 mV at GCE in 0.1 M HCl.

The characterisation of spectra and Voltammograms of silver nanoclay composites (AgNCs) are depicted in Fig. 1.

Figure 1 (a) shows absorption bands difference between the NC and the AgNCs with C-H group at 2800 cm^−1^ and 2900 cm^−1^ for pure nanoclay and absent in spectra of AgNCs suggesting that there is a reaction that took place between silver and nanoclay. This is also indicated by the appearance of a broad O–H peak in AgNCs beyond 3000 cm^−1^, indicating the interaction of the silicate layers of NC and silver nanoparticles as a result of the presence of Van der Waals interacting with the O–H group of NC and the partial positive charge on the silver nanoparticle surface [19, 26]. The C = O intense infra-red stretching absorptions observed around 1600 cm^−1^ in AgNCs is due to the trisodium citrate (TSC) which was used as the reducing agent for silver nanoparticles [19, 27, 28], while the strong Si–O stretching absorption in 1000 cm^−1^ observed for NC only is proof of the successful functionalisation of NC with silver.

The X-ray diffraction peaks in Fig. 1 (b) at 38°, 44°, 64° and 78° corresponding to 111, 200, 220 and 311, observed in the composite and absent in the spectra of the NC, are due to silver as supported by the literature [19, 29, 30], thus proving successful functionalisation of NC with silver [19]. The cyclic voltammogram representation in Fig. 1 (c), revealed the presence of silver in AgNCs through the Ag^+^/Ag couple peaks, which are not appearing in the nanoclay voltammogram. This was accompanied by an increase in current, suggesting that the conductivity of NC was enhanced by the presence of silver [18, 19], Hence, huge current peak observed for silver nanoclay composites as confirmed with differential-pulse voltammetry in Fig. 1 (d).

### Optimisation of Electrode Modification

**Fig. 2:**
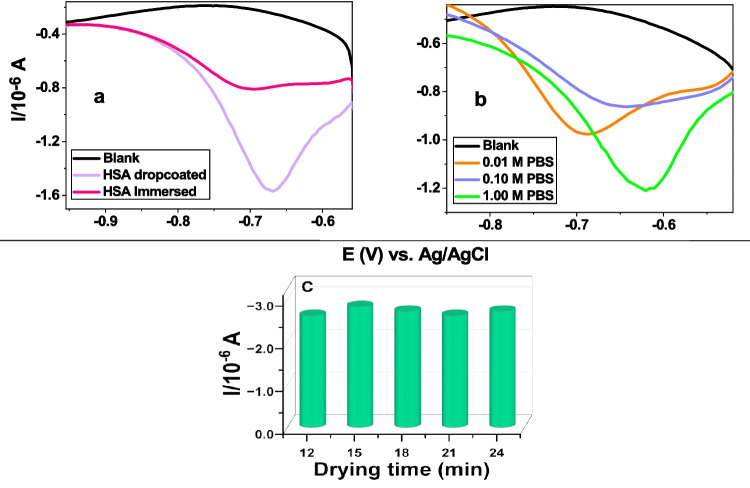
Sensor fabrication optimisation of (**a**) incorporation techniques for HSA (**b**) PBS concentration variation (**c**) HSA drying time for detection of 2.5 μM ZDV with DPV at GCE/AgNCs/HAS.

The fabrication method of HSA, PBS concentration, and drying time of HSA results are shown in Fig. 2. From Fig. 2 (a), the peak potential of ZDV for both dropcoating and immersion method was observed within the potential range of −0.4 V to −1 V, which corresponds to the literature showing the ZDV signal within that potential range [31]. However, enhanced signal with well-defined voltammogram was obtained when HSA was dropcoated compared to when it was immersed. Hence, dropcoating method was considered for further studies. The choice of PBS concentration was assessed with HSA dropcoating method, where the ZDV signal produced by GCE/AgNCs/HSA sensor was seen to increase with increasing concentration of PBS from 0.01 M to 1 M, as can be seen in Fig. 2 (b). PBS is commonly used as a supporting electrolyte in biological systems research due to its nontoxicity to cells. The increase in signal as the concentration of the PBS increases may be due to an increase in ionic strength, thus improving conductivity. The phosphate buffer reduces the pH shifts during the reaction by providing OH^−^, thus ensuring a better catalytic property of the nanoclay composite [32]. Hence, a sharp increase in current was observed which decreased with enhanced drying time as can be seen in Fig. 2 (c). Additionally, appearance of the peak beyond −0.6 V in Fig. 2 (b) is due to the presence of oxygen in the electrolyte.

### Optimisation of Procedure for ZDV Detection

**Fig. 3:**
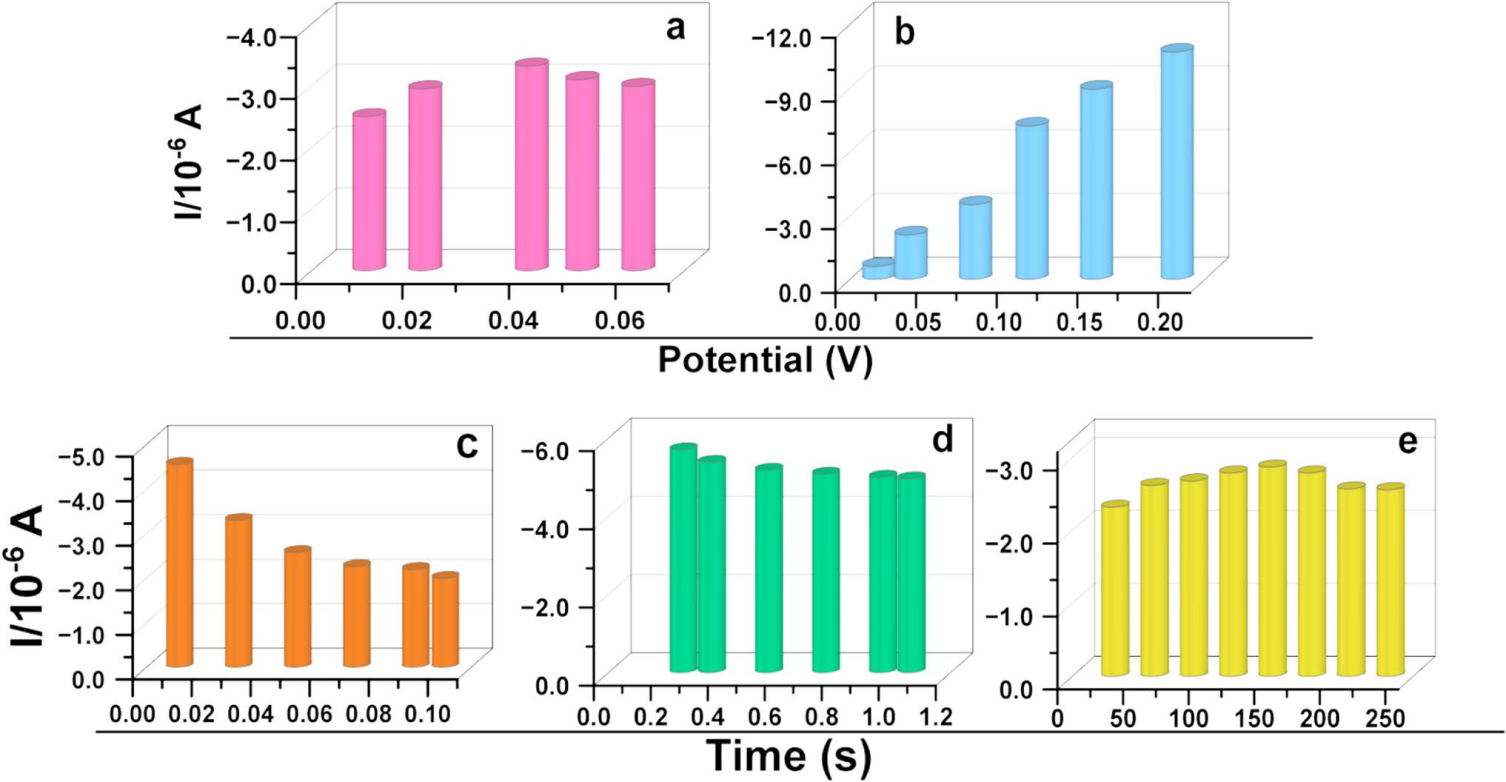
Method optimisation (**a**) step potential (**b**) modulation amplitude (**c**) modulation time (**d**) interval time (**e**) electrolyte stirring time for 2.5 μM ZDV in 1 M PBS, pH 7.04 at GCE/AgNCs/HAS.

The effect of electrolyte stirring time on ZDV signal was studied, where 1 M PBS electrolyte was stirred multiple times, resulting in enhanced ZDV response with increased stirring time as shown in Fig. 3 (e). However, after 2.5 min, the signal decreased leading to the consideration of 2.5 min. Subsequently, varying step potential (Fig. 3a) led to an increase in ZDV signal, with 0.0405 V displaying a greater signal which decreased after. Moreover, the 0.0405 V choice of step potential was used to monitor ZDV response with changes in modulation amplitude (Fig. 3b), where the signal of ZDV was increasing with increasing modulation amplitude. Therefore, 0.075 V was implemented to monitor ZDV response with varying modulation time (Fig. 3c). The results depicted a decrease in current response of ZDV as the modulation time was increased which affected the sensitivity of the sensor. Hence, 0.01 s modulation time was considered. The last parameter investigated was interval time (Fig. 3d) which was monitored from 0.25 to 1.05 s, where the sensitivity of the sensor was affected with increasing interval time. Therefore, 0.25 s was implemented, and all these chosen parameters were used for calibration which will be discussed in the study.

### Mechanism of Electrochemical Process of ZDV

**Fig. 4:**
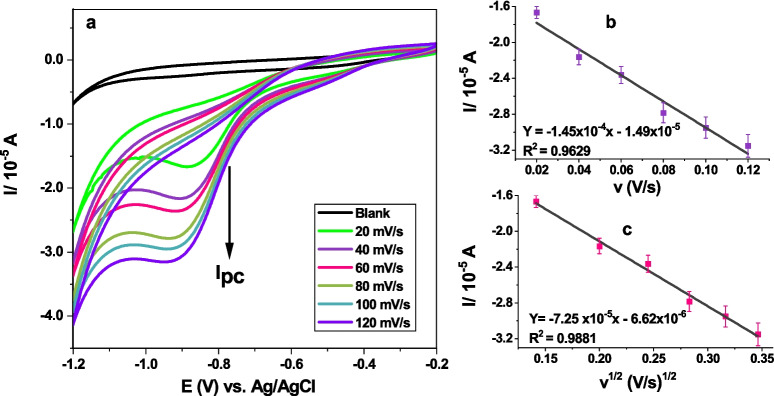
CVs of reduction of ZDV at different scan rates in 1 M PBS at GCE/AgNCs/HSA (**a**), linear plots of ZDV cathodic peak current *versus* (**b**) scan rates and (**c**) square root of scan rates.

The electrochemical kinetics of 0.03 mM ZDV were determined through voltammograms obtained with different scan rates of cyclic voltammetry in 1 M PBS, pH 7.04 as depicted in Fig. 4. From Fig. 4 (a) an increase in cathodic peak current between the potential range of −0.4 V to −1 V which shifts cathodically with increasing scan rate was observed. The corresponding linear plots (Laviron’s and Randles) are represented in Fig. 4 (b, c) respectively.

**Scheme 1:**
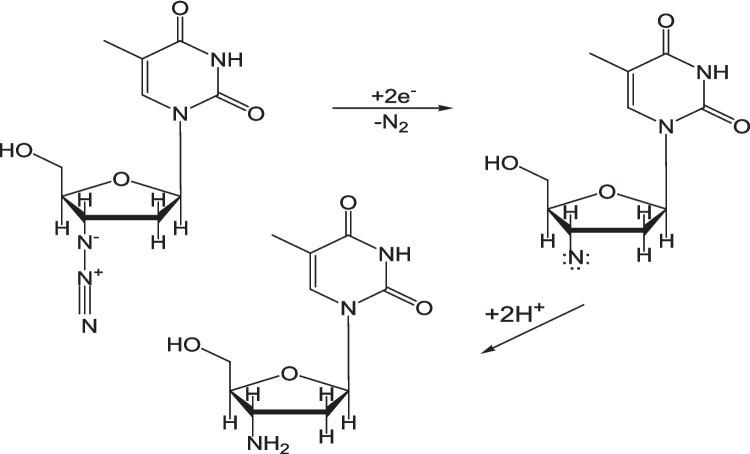
ZDV reduction reaction mechanism.

The ZDV peak shifting cathodically with increasing scan is attributed to involvement of protons in the ZDV reduction reaction [31], as represented in Scheme 1.

The Randles–Sevcik plot has a correlation value of 0.9881 (Fig. 4c) thus indicative that the electroreduction of ZDV is diffusion controlled as previously reported [31], comparing with plot for current vs scan rate (Fig. 4b) with a correlation value of 0.9629. This analysis of Randles–Sevcik plot was applied in assessment of the redox properties of the sensor (GCE/AgNCs/HSA). From the plot Eqs. (1.1–1.3) were used to determine the electrochemical properties of the film, where diffusion coefficient (D) was calculated as 1.55 × 10^–11^ cm^2^ s^−1^ and heterogeneous rate constant (Ks) which was 3.40 × 10^–6^ cm.s^−1^, with two electron transfer process.

1.1 $$\left|{\mathrm{E}}_{\mathrm{p}}-\frac{{\mathrm{E}}_{\mathrm{p}}}{2}\right|=2.20\frac{\mathrm{RT}}{\mathrm{nF}}$$

1.2 $${\mathrm{I}}_{\mathrm{p}}=2.69\times {10}^{5}.{\mathrm{n}}^{3/2}.\mathrm{A}. {\mathrm{ D}}^{1/2}.\mathrm{C}.{\mathrm{v}}^{1/2}$$

1.3 $$\mathrm{Ks}=\frac{{\mathrm{I}}_{\mathrm{p}}}{\mathrm{nFAC}}$$

where E_p_ is the peak potential (V), R is the gas constant (8.314 J.mol^−1^ K^−1^), T is the absolute temperature (kelvin), n is the number of electrons involved, F is the Faraday's constant (96 485.332 C.mol^−1^), I_p_ is the peak current (A), A is the glassy carbon electrode surface area (0.0707 cm^2^), D is the diffusion coefficient (cm^2^.s^−1^), C is the concentration of the electrolyte (mol.cm^−3^), v is the scan rate (V.s^−1^), Ks is the heterogeneous rate constant (cm.s^−1^).

### Calibration

**Fig. 5:**
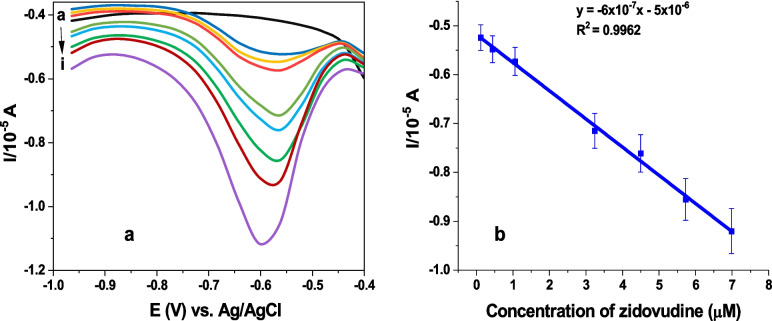
DPVs (**a**) and calibration curve (**b**) of ZDV in 1 M PBS, pH 7.04 at GCE/AgNCs/HSA (parameters: 0.0405 V step potential, 0.075 V modulation amplitude, 0.01 s modulation time, 0.25 s interval time).

The calibration plot (b) in Fig. 5 resulted from voltammograms (a) showing an increased ZDV response with standard addition of ZDV from 0.12 μM to 8.22 μM in 1 M PBS, pH 7.04.

The analytical parameters of GCE/AgNCs/HSA were calculated from the calibration plot. A regression coefficient (R^2^ = 0.9962; n = 7), peak potential of −0.561 V at a linear range of 0.12–6.98 μM were obtained. The Limit of detection (LOD) and Limit of quantification (LOQ) were calculated with equation LOD = 3σ/m and LOQ = 10σ/m, where σ is the standard error of y-intercept, m is the slope of the graph. The results obtained were compared with the previous studies in Table I. The LOD for the developed sensor appeared higher than the one generated with mercury electrode as reported previously. However, due to the toxicity of mercury, the developed sensor can be used as an alternative.

**Table I:** Electrochemical Comparative Studies for ZDV Detection

Method of detection	Working electrode	LOD μM	Linear range μM	References
ASV	Thin-film mercury	0.0025	0.0037—0.29	[4]
SWV	HDME	0.001	0.0005—1	[33]
DPV	HMDE	0.0093	0.93—4.67	[34]
DPV	Co(II)–Cy–PAA graphite paste electrode	1	1 −100	[35]
Amperometry	Ag-NF/M-MWCNT/GC	0.15	0.37—1 496	[31]
CV	Ch@AgNPs/SPGE	1	1—718	[36]
DPV	GCE/AgNCs/HSA	0.3	0.12—6.98	This work

*SWV* Square wave voltammetry; *DPV* Differential pulse voltammetry; *ASV* Anodic stripping voltammetry; *CV* Cyclic voltammetry

### Reproducibility, Repeatability, Stability and Interferences

**Fig. 6:**
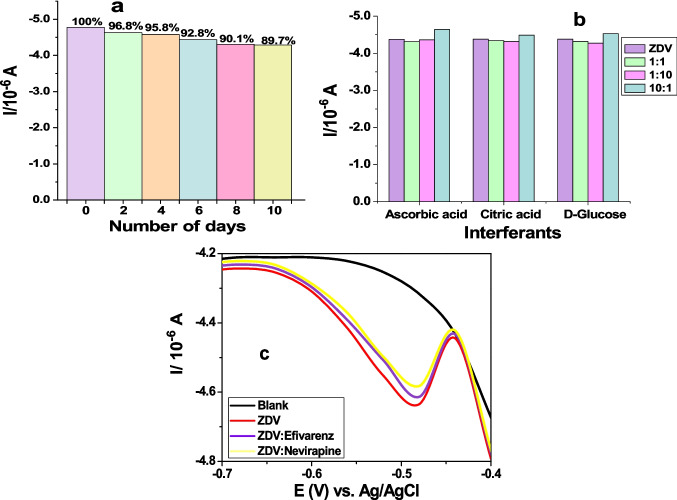
Signal changes over 10 days (**a**) interferants effect of food supplements (**b**) and antiretroviral drugs (**c**).

The reproducibility of the developed sensor was assessed in 1 M PBS, pH 7.04 for detection of 3.24 μM ZDV using DPV. This resulted to a relative standard deviation (RSD) of 5.3% upon five measurements with the same modified electrode (GCE/AgNCs/HSA). The repeatability of the sensor on the other hand resulted to an acceptable RSD value of 1.59% which was obtained from five successive measurements of 3.24 μM ZDV. Moreover, the sensor was tested for stability over a period of 10 days as shown in Fig. 6 (a), where there was a slight decrease in ZDV current signal to HSA with increasing number of days.

The stability is aligned to the acclaimed nanoclay sensor stability that is due to its chemical inertness, ion exchange ability, swelling, mechanical and thermal stabilities. Hence, the reason for their becoming versatile and non-expensive matrices used in a variety of electrochemical studies [21, 37].

The Interference study of ZDV was assessed in the presence of food supplements such as ascorbic acid, citric acid, and D-glucose (Fig. 6b), and in the presence of other ARV drugs namely efavirenz and nevirapine (Fig. 6c). The impact of these interferants on the ZDV response show a slight decrease in the signal of pure ZDV in both Fig. 6 (b) and (c). However, when ZDV was present in high concentration in the electrolyte with less concentration of the food supplements (10:1), the greater signal of ZDV was detected. The sensor was also able to detect ZDV even when the interferants were present in 10-folds in the solution (1:10). This suggests good selectivity due to the interaction of the analyte with the biological recognition element (HSA) through electrostatic, intercalation and groove binding, forming a measurable signal [11, 38].

### Recovery Studies of ZDV in Spiked Urine Samples

The recovery studies were done by diluting urine with different volumes of PBS and spiked with 1.12 μM ZDV in the ratios of the dilutions prepared i.e. 1:4, 1:9 and 1:14 Urine:PBS. The recoveries determined were 116%, 95% and 105% respectively, as shown in Table II.

**Table II:** ZDV Drug Spiking/Recovery Studies

ZDV	Urine:PBS		
	1:4	1:9	1:14
[ZDV] _added_ (μM)	1.12	1.12	1.12
[ZDV] _found_ (μM)	1.30	1.06	1.18
	%Recovery	95	105

### Quantification of ZDV in Commercial Tablets

**Fig. 7:**
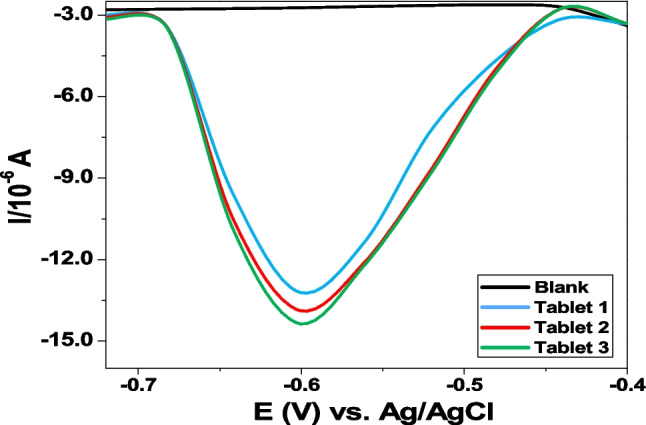
DPV of the first run of the three commercial tablets at GCE/AgNCs/HSA in 1 M PBS, pH 7.04.

The detection of ZDV in three commercial tablets was done and the resulting currents are shown in Fig. 7.

The currents generated together with the calibration equation (y = −6 × 10^–7^ x −5 × 10^–6^) were used to calculate the unknown concentration of ZDV in each solution, where x is the unknown concentration of the analyte in solution, and y is the current measured. Using the molar mass of ZDV and the concentrations calculated, the content of ZDV in each tablet was obtained to be 300.8, 301.6, and 301.8 mg respectively, resulting in average mean of 301.3 mg ZDV of all three tablets which is comparative to the commercial tablet stated amount (300 mg), with 1.9% RSD less than 5%, implying that the data measured is precise.

## Conclusions

A sensitive, selective, reproducible and accurate electroanalytical technique using DPV for detection and quantification of ZDV using GCE/AgNCs/HSA sensor was fabricated, optimised and its application was assessed. The electrode modification optimum conditions were HSA drop coating, with 15 min drying time using 1 M PBS electrolyte. While the best procedure regarding the step potential, modulation amplitude, modulation time, interval time, and electrolyte stirring time were found to be 0.0405 V, 0.075 V, 0.01 s, 0.25 s, and 2.5 min analysis respectively. These conditions were used to determine the electrochemical kinetics of ZDV and analytical parameters (linear range, detection limit and quantification limit). The developed sensor applicability was validated showing good repeatability, recovery, reproducibility and acceptable interference results. Furthermore, the sensor maintained the stability of ZDV current signal to HSA for 10 days. This sensor was used to analyse real pharmaceutical tablets, where the three tablets analysed values were closer to the stated amount despite the weight variations. This sensor is an alternative low-cost analysis for detection and quantification of ZDV that can be applied in real biological and pharmaceutical sample analysis. Thus, ideal for rural clinics for monitoring quality of drugs storage and ensuring good quality products for patients at low cost.
